# Identity-Based Encryption with Filtered Equality Test for Smart City Applications

**DOI:** 10.3390/s19143046

**Published:** 2019-07-10

**Authors:** Yang Ming, Erxiu Wang

**Affiliations:** School of Information Engineering, Chang’an University, Xi’an 710064, China

**Keywords:** smart healthcare, identity-based encryption, filtered equality test, random oracle model

## Abstract

With the growth of the urban population, the rapid development of smart cities has become the focus of urban regional development. Smart medical care is an indispensable part of smart city construction, which promotes the development of the medical industry. However, the security of data and timely service are the current problems faced by intelligent medical systems. Based on the public key encryption with filtered equality test and identity-based cryptography, an identity-based encryption with the filtered equality test (IBE-FET) is proposed for smart healthcare, in which a data receiver can use the private key and the message set to generate a warrant and send it to the cloud server. A cloud server can verify the equality between ciphertexts without decryption and check whether the encrypted message belongs to the same message set. Furthermore, the security analysis shows that the proposed scheme satisfies one-way security against the chosen identity and ciphertext attack in the random oracle model under the computational bilinear Diffie-Hellman assumption. The performance comparison shows that the scheme is feasible and practical in real life.

## 1. Introduction

The concept of the smart city (SC) [1] emerges in the context in which the current global power supply and consumption trends are socially, environmentally and economically unsustainable. It refers to an urban transformation which, with the use of the latest information and communications technologies (ICT), improves cities’ efficiency. Currently, more and more people live in cities and every person uses more than five devices to access the Internet. Thus, the various embedded devices are integrated with urban infrastructure to optimize daily life of citizens.

**Figure 1:**
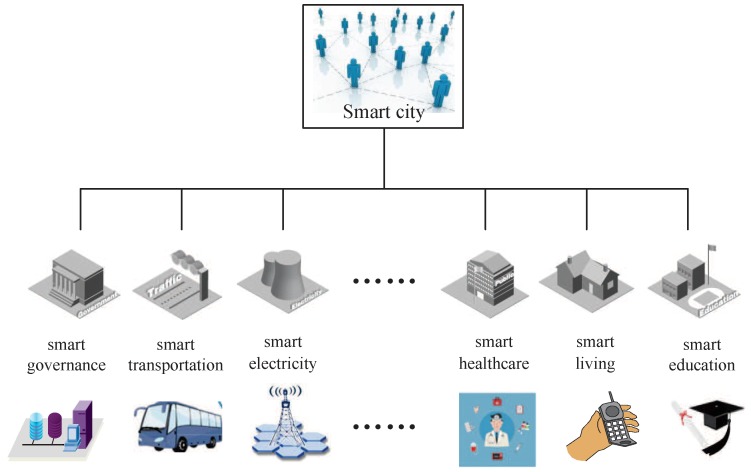
Representative smart city applications.

Recently, with the rapid development of the Internet of Things (IoT) [2] and ICT, the applications of the smart city [3] are on the rise, which can enhance the life quality of citizens. Representative smart city applications are given in Figure 1, which benefit the city and people in a variety of aspects: economy, education, healthcare, and living. Meanwhile, the smart city has a new, complete level of effectiveness, sustainability and efficiency.

The main goal of the smart city is to greatly improve quality of life. Nevertheless, the security and privacy problems are of great importance to the users in the smart city [4,5,6]. Progress in the IoT and cloud computing technology is driving the development of smart systems to support and improve healthcare system. However, the current healthcare system is faced with a series of challenges in providing low cost health care services. Besides, it is difficult for patients in some areas to obtain a timely healthcare services due to poor medical conditions. As a result, smart healthcare [7,8] has emerged recently as the key component of a new generation healthcare network. The so-called smart healthcare is to improve the efficiency of biomedical systems and healthcare infrastructures through various entities and technologies, including smart sensors, wearable devices, ICT and more [9].

In the smart healthcare system, patients are paying more and more attention to the security of private information. Zhang et al. [10,11,12,13] has done in-depth research and proposed privacy-preserving access control systems by adopting attribute-based encryption techniques to improve the security of smart healthcare. However, the techniques are complex and unfeasible in practice. To save storage space and protect the user’s privacy, the sensitive information must be stored in the untrusted healthcare cloud servers in an encrypted form. However, given some ciphertexts, no one can distinguish the relationships among the ciphertexts without decryption. Searchable encryption (SE) [14,15,16] is a practical and promising solution to this problem. To provide the capability for searching in the ciphertexts, the public key encryption with keyword search (PKE-KS) schemes [17,18,19,20,21,22] were proposed, which is one practical implementation of SE. However, the PKE-KS schemes have one weakness that the ciphertexts are generated by the same public keys and therefore it is not applicable to some scenarios. To solve this problem, the public key encryption with equality test (PKE-ET) schemes [23,24,25,26,27,28,29,30,31] were put forward, which allowed equality tests made on the ciphertexts by different public keys as well as the same public keys. To alleviate the storage cost of certificates, identity-based encryption with equality test (IBE-ET) schemes [32,33] were proposed. Along with research, to make fine-grained authorization more flexible and inspired by the idea of attribute-based encryption, the attribute-based encryption with equality test (ABE-ET) schemes [34,35,36,37] were presented.

To provide more flexible equality testing to satisfy different requirements, Huang et al. presented the public key encryption with filtered equality test (PKE-FET) schemes [38,39], in which only a few selected message sets can be equality tested. An authorized user can determine not only whether two ciphertexts contain the same plaintext (without decryption) but also whether the plaintext belongs to the message set.

**Figure 2:**
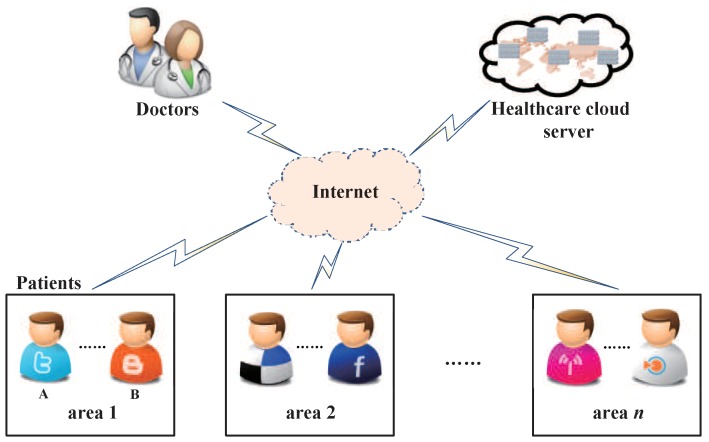
A practical application scenario of identity-based encryption with filtered equality test (IBE-FET).

In this paper, we integrate the identity-based cryptography [40] into PKE-FET to propose a new concept of identity-based encryption with the filtered equality test (IBE-FET) for smart healthcare. A practical application scenario using IBE-FET is shown in Figure 2.

In the smart healthcare system, there are three parties: doctors, the healthcare cloud server (HCS) and patients, where the patients are distributed in different areas. To ensure the privacy of patients, the sensitive data is encrypted during transmission. It is desired that the healthcare providers optimize the distribution of family doctors, and thus they need to search for the encrypted information. With the assumption that patients A and B with the same symptoms belong to area 1, A encrypts his privacy information (symptom and area) under the identity $ID_{A}$ and the doctor’s identity $ID_{D}$, and transmits the tuple $\left\{ID_{A},\text{IBE-FET}\left(ID_{D},ID_{A},\;\mathrm{symptom},\;\mathrm{area}\;1\right)\right\}$ to HCS. Additionally, A generates a warrant $w_{A}$ and transmits to HCS. B transmits $\left\{ID_{B},\text{IBE-FET}\left(ID_{D},ID_{B},\;\mathrm{symptom},\;\mathrm{area}\;1\right)\right\}$ and $w_{B}$ to HCS in the same way. Upon obtaining these data, the HCS could determine and search whether A and B are distributed in the same areas and have the same symptom. However, there is no knowledge what the real areas and symptom are. Then, the HCS sends the search result to the patients A and B, respectively, which allows them to share their medical experience with each other. Most important of all, the HCS can investigate the cause of the disease and arrange family doctors reasonably to improve the efficiency of healthcare. The above scenario can be extended to multi-user scenarios. For instance, more patients can get the warrant and send it to the HCS along with the requests and obtain feedback, indicating whether there are any patients belonging to the same area who have the same symptom features.

Besides, the IBE-FET scheme can also be applied to the smart grid system [41,42], which contains electricity suppliers, a power system cloud server and users. To protect the privacy and enhance the power quality of users, the privacy information (e.g., power consumers and location) is generally transmitted in encrypted form. Based on IBE-FET, the power system cloud server can determine and search whether there are any users belonging to the same area that have the same feature (e.g., power flow and peak loading). Then, they send the search result to the electricity suppliers for improvement of the power distribution and optimization of the power flow.

### 1.1. Our Contributions

This paper proposes an identity-based encryption with the filtered equality test (IBE-FET). The main contributions are summarized as follows:

- Based on secret sharing and bilinear pairing, an IBE-FET scheme is proposed, which does not use the certificate verification to solve the problems of certificate management.
- The security analysis indicates that the IBE-FET scheme is one-way secure against the chosen identity and ciphertext attack (OW-ID-CCA) based on the computational bilinear Diffie-Hellman assumption in the random oracle model.
- The performance analysis shows that the IBE-FET scheme achieves the function of a filtered equality test and a higher efficiency in terms of communication cost than the related scheme [39], and therefore the proposed scheme is more suitable for smart healthcare systems.

### 1.2. Organization

The organization of this paper is as follows: We will briefly discuss related work in Section 2 and review some preliminaries in Section 3; in Section 4, we introduce the framework of IBE-FET; a concrete IBE-FET scheme is put forward in Section 5; Section 6 proposes a formal security proof; comparison and performance evaluations are described in Section 7; and Section 8 concludes this paper.

## 2. Related Works

The concept of public key encryption with the keyword search (PKE-KS) was first put forward by Boneh et al. [17]. In PKE-KS, each user can use their private key to generate a token for a keyword and send the token to the tester. Upon receiving the token, the tester can determine the equality of ciphertexts. Then, some interesting extension schemes [18,19,20,21,22] were proposed to satisfy various requirements.

PKE-KS aims at testing the keyword’s equality using a given trapdoor. However, it is not suitable for an equality test on ciphertexts by different public keys. In order to solve this problem, Yang et al. [23] proposed public key encryption with the equality test (PKE-ET). The so-called “equality test (ET)” refers to an authorized user who can verify the equality of two ciphertexts encrypted by different public keys, while the decryption keeps unavailable. However, in the PKE-ET scheme, anyone has the ability to execute the equality test without any authorization. As a fundamental security service, the authorization mechanism becomes increasingly important in modern smart system. The hierarchical key assignment techniques [43,44,45,46] were presented, which can provide fine-grained authentication and access control for the user. In order to mitigate the potential vulnerabilities and protect the user’s privacy, Tang et al. [24] integrated the fine-grained authorization mechanism into PKE-ET. In this scheme, two users require cooperation to generate the token by running the authorization algorithm and send this token to the tester, with the tester authorized to verify the equality between the ciphertexts. In addition, Tang et al. [25] introduced the concept of coarse-grained authorization scheme, in this system, every user independently generates the token by running the authorization algorithm and sends it to the tester, who executes the equality test from their ciphertexts. In 2012, Tang [26] expanded [24] to a two-proxy agents setting, where two proxies require cooperation to perform the equality test. Lu et al. [27] introduced a stronger security model for PKE-ET to meet the different demands. In 2015, the public key encryption with the delegated equality test scheme (PKE-DET) was proposed by Ma et al. [28] and in this scheme every user can generate the delegation token independently for the cloud server. Different from PKE-DET, Huang et al. [29] introduced an efficient public key encryption with the authorized equality test (PKE-AET), a provision of two kinds of warrants (recipient warrants and ciphertext warrants) and allowance of the authorized users to use warrants to execute the equality test on two ciphertexts encrypted by different public keys. To satisfy various requirements, the public key encryption supporting equality test and flexible authorization (PKE-ET-FA) was proposed by Ma et al. [30]. In this scheme, four types of authorization were presented to strengthen the user privacy protection. However, it is inefficient due to using bilinear pairings. In 2016, Lin et al. [31] proposed an efficient PKE-ET-FA scheme without using bilinear pairing, which was more suitable for practice. In order to solve the certificate management problem, the identity-based encryption with equality test (IBE-ET) [32,33] was presented. To determine the equality of two ciphertexts encrypted under different access policies, the attribute-based encryption with equality test schemes (ABE-ET) [34,35,36,37] were put forward.

For making the equality test more flexible, based on bilinear pairing and secret sharing, Huang et al. [38,39] proposed the public key encryption with the filtered equality test (PKE-FET). In these schemes, the receiver selects *n* messages as a set Ω, and then the receiver can use a private key and Ω to generate the warrant *w* and sends this warrant to someone, who can execute the equality test without decryption.

The PKE-FET scheme needs certification authority to ensure the authenticity of public keys; however, it is worth noting that the problems of certificate management arise. Accordingly, inspired by the concept of identity-based cryptography [40,47,48], we presented an identity-based encryption with the filtered equality test scheme (IBE-FET), simplifying the certificate management of PKE-FET.

## 3. Preliminaries

This section introduces some preliminaries, including bilinear pairing, secret sharing and security assumption.

### 3.1. Bilinear Pairing

Let $G_{1}$, $G_{T}$ be two cyclic groups of prime order *q*, and *g* is a generator of $G_{1}$. $e:G_{1}\times G_{1}\to G_{T}$ is a bilinear pairing if the following three properties hold:

- **Bilinearity**: For all $u,v\in G_{1}$ and $a,b\in Z_{q}^{*}$, where $e\left(u^{a},v^{b}\right)=e{\left(u,v\right)}^{ab}$.
- **Non-degeneracy**: e(g,g)≠1.
- **Computability**: It is an efficient algorithm to compute e(u,v) for all $u,v\in G_{1}$.

### 3.2. Secret Sharing

The idea of secret sharing is introduced in [49], with a secret value *k* assigned to *n* users. A trusted party holds *k* and randomly picks t−1 numbers $r_{1},r_{2},\cdot \cdot \cdot ,r_{t-1}$ form *t* points on a 2-dimensional plane, which are $\left\{\left(0,k\right),\left(1,r_{1}\right),\cdot \cdot \cdot ,\left(t-1,r_{t-1}\right)\right\}$. According to these points, there is only one polynomial function ψ with t−1 degree determined. Then, the trusted party computes the points (i,ψ(i)) for user i∈[t,n], in which all the points satisfy $y_{i}=\psi \left(i\right)$. By distributing these points, it formalizes a *t*-out-of-*n* secret sharing scheme. Therefore, as for any *t* or more than *t* users, it can reconstruct the polynomial function ψ and obtain the secret value *k* by computing k=ψ(0), but if less than *t* users, it cannot rebuild the secret value *k*.

### 3.3. Assumption

**Computational Bilinear Diffie-Hellman (CBDH) Problem**: Let *g* be the generator of $G_{1}$ and $a,b,c\in Z_{q}^{*}$ be chosen at randomly. Given a tuple $\left(g,g^{a},g^{b},g^{c}\right)\in G_{1}$, the task of CBDH problem is to compute $e{\left(g,g\right)}^{abc}\in G_{T}$.

The probability of the algorithm A in solving the CBDH problem is defined as

$$Adv_{A}^{CBDH}=\mathrm{Pr}\left[A\left(g,g^{a},g^{b},g^{c}\right)=e{\left(g,g\right)}^{abc}\right]⩽\varepsilon .$$

**Computational Bilinear Diffie-Hellman (CBDH) Assumption**: The CBDH assumption holds if for any polynomial-time algorithm A solves the CBDH problem with the negligible probability.

## 4. Framework of IBE-FET

The system model, syntax and security model are described in the following sections.

### 4.1. System Model

**Figure 3:**
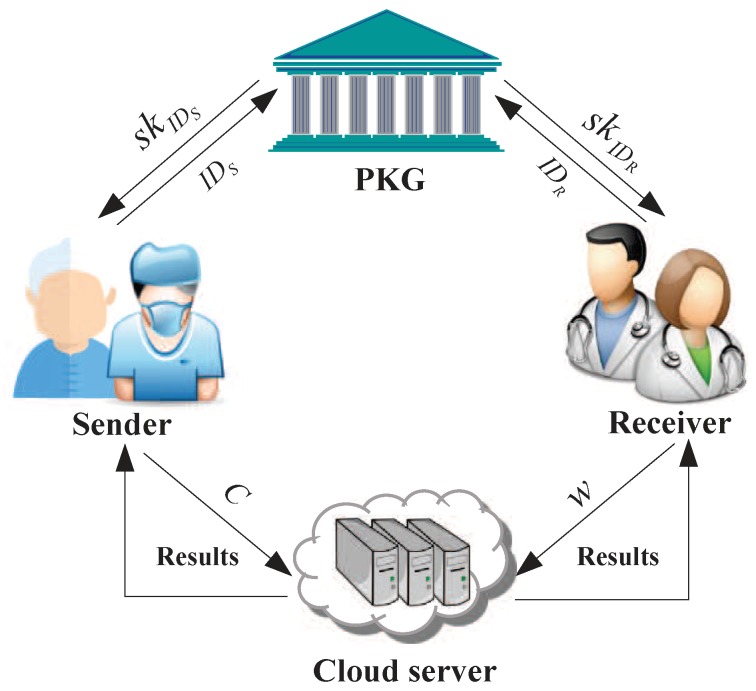
System model for IBE-FET.

The system model of IBE-FET includes four parts: private key generator (PKG), sender (patient), receiver (doctor) and the cloud server, as illustrated in Figure 3. All ciphertexts are generated by the senders under the receiver’s identity and stored in the cloud server. The PKG’s task is to generate the private keys for the users (senders and receivers) secretly. To compare the ciphertexts, the receiver generates the corresponding warrant using its private key and the message set, sending it to the cloud server; wherein the warrant denotes the trapdoor of authentication. As a result, with the warrant, the cloud server is able to verify the equality between the ciphertexts without decryption and check whether the message belongs to the message set. The work of each part is described in more details below:

- **PKG**: It is responsible for generating the master key msk and the private key $sk_{ID}$, and then keeps msk by itself and sends $sk_{ID}$ to the sender and receiver through a secure way.
- **Sender (patient)**: The sender encrypts their private date under the receiver’s identity $ID_{R}$ to generate the ciphertext *C* and stores it in the cloud server.
- **Receiver (doctor)**: Upon receiving the private key $sk_{ID_{R}}$ from PKG, the receiver generates the warrant *w* and sends it to the cloud server. It is noted that the receiver can use the private key to decrypt the ciphertext at any time.
- **Cloud server**: With the warrant, the cloud server is in charge of executing the filtered equality test and returns a query result.

**Figure 4:**
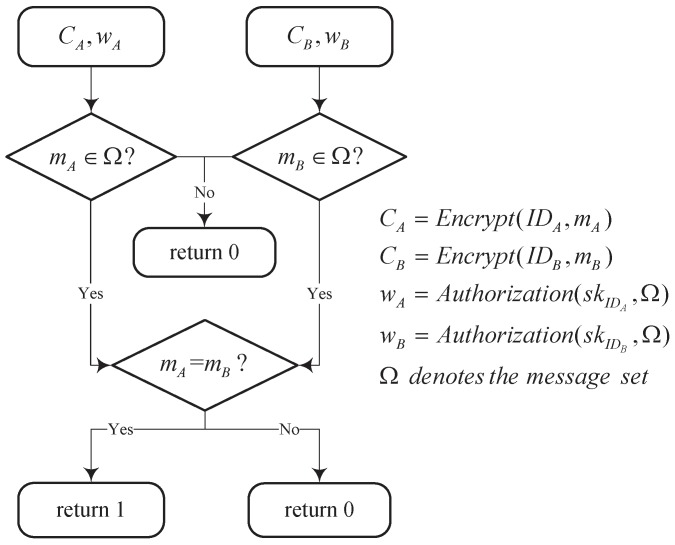
Flow chart of FET.

The detail data flow of the filtered equality test (FET) is described in Figure 4.

### 4.2. Syntax

The IBE-FET scheme consists of the following six algorithms: setup, extract, encrypt, decrypt, authorization and filtered equality test. Let Δ denote message space and Ω⊆Δ denote the message set.

**Setup**: Taking a security parameter *k* as input, this algorithm outputs the master key msk and the system parameters PP.

**Extract**: Taking the master key msk and the identity ID as input, this algorithm outputs the private key $sk_{ID}$.

**Encrypt**: Taking the system parameters PP, the plaintext m∈Δ and the identity ID as input, this algorithm outputs the ciphertext *C*.

**Decrypt**: Taking the system parameters PP, the ciphertext *C* and the private key $sk_{ID}$ as input, this algorithm outputs the corresponding plaintext *m*.

**Authorization**: Taking the system parameters PP, the identity ID, the private key $sk_{ID}$ and the message set Ω as input, this algorithm outputs the warrant $w_{ID}$.

**Filtered equality test**: Taking the system parameters PP, the ciphertexts $C_{A}$ and $C_{B}$, the warrants $w_{ID_{A}}$ and $w_{ID_{B}}$ as input, this algorithm returns 1 if $m_{A}\in \Omega$, $m_{B}\in \Omega$ and $m_{A}=m_{B}$. Otherwise, it returns 0.

For the property of consistency, the following conditions must be satisfied.

**Correctness**: When $sk_{ID}$ is generated by the **Extract** algorithm given ID, then, for all m∈Δ, $\mathrm{Pr}[\mathrm{Decrypt}\left(\mathrm{Encrypt}\left(ID,m\right),sk_{ID}\right)=m]=1$.

**Perfect consistency**: When $w_{ID_{A}}$ and $w_{ID_{B}}$ are generated by the **Authorization** algorithm given $ID_{A}$, $ID_{B}$ and Ω, then, for all $m_{A}\in \Omega$, $m_{B}\in \Omega$ and $m_{A}=m_{B}$, the filtered equality test algorithm must return 1.

**Computational soundness**: When $w_{ID_{A}}$ and $w_{ID_{B}}$ are generated by the **Authorization** algorithm given $ID_{A}$, $ID_{B}$ and Ω, then, for all $m_{A}\in \Omega$, $m_{B}\in \Omega$ and $m_{A}\neq m_{B}$, the probability that the filtered equality test algorithm returns 1 is negligible.

### 4.3. Security Model

The security of IBE-FET needs to satisfy one-way security against the chosen identity and ciphertext attack (OW-ID-CCA), which is defined by an interactive game between a challenger C and an adversary A.

**Setup**: C generates the master key msk and the system parameters $PP_{IBE-FET}$ by running the **Setup** algorithm. Then C sends $PP_{IBE-FET}$ to A and keeps msk by itself.

**Phase 1**: A makes the following queries for polynomial number of times.

- **Hash *H* queries**: A submits a query, then C returns a random value to A.
- **Private key queries**: A submits the identity $ID_{j}$ to C, then C runs the **Extract** algorithm and returns the private key $sk_{ID_{j}}$ to A.
- **Decryption queries**: A submits the identity $ID_{j}$ and the ciphertext $C_{j}$ to C, then C runs the **Extract** algorithm to obtain $sk_{ID_{j}}$ and runs the **Decrypt** algorithm to return the plaintext $m_{j}$ to A.
- **Authorization queries**: A submits the identity $ID_{j}$ and the message set $\Omega_{j}$ to C, then C runs the **Extract** algorithm to obtain $sk_{ID_{j}}$ and runs the **Authorization** algorithm to return the warrant $w_{ID_{j}}$ to A.

**Challenge**: A submits a challenge identity $ID^{*}$ to C, where $ID^{*}$ does not appear in private key queries in **Phase 1**. C randomly chooses a plaintext $m^{*}\in \Delta$ and sets $C^{*}$ be the challenge ciphertext. Finally, C sends $C^{*}$ to A.

**Phase 2**: Similar to **Phase 1**.

- **Hash *H* queries**: C responds as in **Phase 1**.
- **Private key queries**: If $ID_{j}\neq ID^{*}$, C responds as in **Phase 1**. Otherwise, C returns ⊥.
- **Decryption queries**: If $\left(ID_{j},C_{j}\right)\neq \left(ID^{*},C^{*}\right)$, C responds as in **Phase 1**. Otherwise, C returns ⊥.
- **Authorization queries**: C responds as in **Phase 1**.

**Guess**: A outputs a guess $m^{\prime }$ and wins the above game if $m^{\prime }=m^{*}$.

The advantage of A winning the above game is defined as

$$Adv_{IBE-FET,\;A}^{OW-ID-CCA}=\mathrm{Pr}\left[m^{\prime }=m^{*}\right].$$

**Definition** **1.** *The IBE-FET scheme is OW-ID-CCA security if for any adversaries A, $Adv_{IBE-FET,\;A}^{OW-ID-CCA}$ is negligible.*

Next, the security of the public key encryption (PKE) scheme (which will be mentioned later) needs to satisfy one-way security against the chosen ciphertext attack (OW-CCA), which is defined by an interactive game between a challenger C and an adversary A.

**Setup**: C generates the private key sk and the system parameters $PP_{PKE}$ by running the **Setup** algorithm. Then C sends $PP_{PKE}$ to A and keeps sk by itself.

**Phase 1**: A makes the following queries for polynomial number of times.

- **Hash *H* queries**: A submits a query, then C returns a random value to A.
- **Decryption queries**: A submits the ciphertext $C_{i}$ to C, then C runs the **Decrypt** algorithm and returns the plaintext $m_{i}$ to A.

**Challenge**: C randomly chooses a challenge plaintext $m^{*}\in \Delta$ and runs the **Encrypt** algorithm to obtain the challenge ciphertext $C^{*}$. Finally, C sends $C^{*}$ to A.

**Phase 2**: Similar to **Phase 1**.

- **Hash *H* queries**: C responds as in **Phase 1**.
- **Decryption queries**: If $C_{i}\neq C^{*}$, C responds as in **Phase 1**. Otherwise, C returns ⊥.

**Guess**: A outputs a guess $m^{\prime }$ and wins the above game if $m^{\prime }=m^{*}$.

The advantage of A wining the above game is defined as

$$Adv_{PKE,\;A}^{OW-CCA}=\mathrm{Pr}\left[m^{\prime }=m^{*}\right].$$

**Definition** **2.** *The PKE scheme is OW-CCA security if, for any adversaries A, $Adv_{PKE,\;A}^{OW-CCA}$ is negligible.*

## 5. The Proposed Scheme

In this section, a detailed construction of IBE-FET is proposed.

- **Setup**: Given a security parameter *k*, the PKG executes as follows:
  (1) Chooses a bilinear pairing: $e:G_{1}\times G_{1}\to G_{T}$, where $G_{1}$ and $G_{T}$ are two cyclic groups with prime order *q*, *g* is a generator of $G_{1}$.
  (2) Randomly picks $u,s_{0},s_{1},\cdot \cdot \cdot ,s_{n}\in Z_{q}^{*}$ and computes $U=g^{u}$, $S_{0}=g^{s_{0}}$, $S_{1}=g^{s_{1}}$, ···, $S_{n}=g^{s_{n}}$.
  (3) Chooses four one-way hash functions $H_{1}:{\left\{0,1\right\}}^{*}\to G_{1}$, $H_{2}:{\left\{0,1\right\}}^{l_{1}}\to Z_{q}^{*}\;$, $H_{3}:G_{T}\to {\left\{0,1\right\}}^{l_{1}+l_{2}}$, $H_{4}:{\left\{0,1\right\}}^{l_{1}}\to G_{T}$, where $l_{1}$ is the length of the message and $l_{2}$ is the length of $Z_{q}^{*}$.
  The system parameters are $PP_{IBE-FET}=\left\{e,q,G_{1},G_{T},g,U,S_{0},S_{1},\cdot \cdot \cdot ,S_{n},H_{1},H_{2},H_{3},H_{4}\right\}$ and the master key are $msk=\{u,s_{0},s_{1},\cdot \cdot \cdot ,s_{n}\}$.
- **Extract**: Given the identity ID and the master key $u,s_{0},s_{1},\cdot \cdot \cdot ,s_{n}$, PKG computes $h_{ID}=H_{1}\left(ID\right)$ and the private key $sk_{ID}=\left\{h_{ID}^{u},h_{ID}^{s_{0}},h_{ID}^{s_{1}},\cdot \cdot \cdot ,h_{ID}^{s_{n}}\right\}$.
- **Encrypt**: Given the message *m* and the identity ID, the sender executes as follows:
  (1) Randomly chooses $r,t\in Z_{q}^{*}$.
  (2) Computes $h_{ID}=H_{1}\left(ID\right)$, $h=H_{2}\left(m\right)$, $S=\prod_{i=0}^{n}S_{i}^{rh^{i}}$,
    $C_{1}=\left\{C_{1,0}=g^{r},C_{1,1}=g^{rh},\cdot \cdot \cdot ,C_{1,n}=g^{rh^{n}}\right\}$,
    $C_{2}=g^{t}$,
    $C_{3}=\left(m||r\right)⊕H_{3}\left(e{\left(h_{ID},U\right)}^{t}\right)$,
    $C_{4}=e\left(h_{ID},S\right)\cdot H_{4}\left(m\right)$.
  The ciphertext is $C=\{C_{1},C_{2},C_{3},C_{4}\}$, where $C_{1}=\left(C_{1,0},C_{1,1},\cdot \cdot \cdot ,C_{1,n}\right)$.
- **Decrypt**: Given the ciphertext *C* and the private key $sk_{ID}$, the receiver executes as follows:
  (1) Computes $C_{3}⊕H_{3}\left(e\left(h_{ID}^{u},C_{2}\right)\right)=m\left|\right|r$ and $h=H_{2}\left(m\right)$.
  (2) Verifies
    $$C_{1,i}=g^{rh^{i}}\;\mathrm{and}\;C_{4}=\prod_{i=0}^{n}e(h_{ID}^{s_{i}},C_{1,i})\cdot H_{4}\left(m\right)$$
    for all i∈[0,n]. If holds, it outputs *m*. Otherwise, it outputs ⊥.
- **Authorization**: Given the message set $\Omega =\{m_{1},m_{2},\cdot \cdot \cdot ,m_{n}\}$ and the private key $sk_{ID}=\left\{h_{ID}^{s_{0}},h_{ID}^{s_{1}},\cdot \cdot \cdot ,h_{ID}^{s_{n}}\right\}$, the receiver performs the following steps:
  (1) Computes a *n*-degree polynomial function $f\left(x\right)=\prod_{i=1}^{n}\left(x-H_{2}\left(m_{i}\right)\right)=\sum_{i=0}^{n}a_{i}x^{i}$ and obtains the coefficient $a_{0},a_{1},\cdot \cdot \cdot ,a_{n}$.
  (2) Computes $w_{ID,i}=h_{ID}^{s_{i}}\cdot h_{ID}^{a_{i}}$ for all i∈[0,n] and sends the warrant $w_{ID}=\left\{w_{ID,\;0},w_{ID,\;1},\cdot \cdot \cdot ,w_{ID,n}\right\}$ to the cloud server.
- **Filtered equality test**: Given two ciphertexts $C_{A}=\left\{C_{A,1}=\left(C_{A,1,0},C_{A,1,1},\cdot \cdot \cdot ,C_{A,1,n}\right),C_{A,2},C_{A,3},C_{A,4}\right\}$ and $C_{B}=\left\{C_{B,1}=\left(C_{B,1,0},C_{B,1,1},\cdot \cdot \cdot ,C_{B,1,n}\right),C_{B,2},C_{B,3},C_{B,4}\right\}$, two warrants $w_{ID_{A}}=\left\{w_{ID_{A},\;0},w_{ID_{A},\;1},\cdot \cdot \cdot ,w_{ID_{A},\;n}\right\}$ and $w_{ID_{B}}=\left\{w_{ID_{B},\;0},w_{ID_{B},\;1},\cdot \cdot \cdot ,w_{ID_{B},\;n}\right\}$, the cloud server executes as follows:
  (1) Computes $z_{A}=\frac{C_{A,4}}{\prod_{i=0}^{n}e(C_{A,1,i},w_{ID_{A},\;i})}$ and $z_{B}=\frac{C_{B,4}}{\prod_{i=0}^{n}e(C_{B,1,i},w_{ID_{B},\;i})}$.
  (2) Checks whether $z_{A}=z_{B}$ or not. It outputs 1 if $z_{A}=z_{B}$, which means $m_{A}\in \Omega$, $m_{B}\in \Omega$ and $m_{A}=m_{B}$. Otherwise, it outputs 0.

**Correctness**: The decryption algorithm computes

$$\begin{array}{c} C_{3}⊕H_{3}\left(e\left(h_{ID}^{u},C_{2}\right)\right)\\ =\left(m||r\right)⊕H_{3}\left(e{\left(h_{ID},U\right)}^{t}\right)⊕H_{3}\left(e\left(h_{ID}^{u},g^{t}\right)\right)\\ =\left(m||r\right)⊕H_{3}\left(e{\left(h_{ID},g^{u}\right)}^{t}\right)⊕H_{3}\left(e\left(h_{ID}^{u},g^{t}\right)\right)\\ =m||r \end{array}$$

Then, let $h=H_{2}\left(m\right)$, it checks both $C_{1,i}=g^{rh^{i}}$ and $C_{4}=\prod_{i=0}^{n}e(h_{ID}^{s_{i}},C_{1,i})\cdot H_{4}\left(m\right)=\prod_{i=0}^{n}e(h_{ID}^{s_{i}},g^{rh^{i}})\cdot H_{4}\left(m\right)=e{\left(h_{ID},g\right)}^{r\sum_{i=0}^{n}s_{i}h^{i}}\cdot H_{4}\left(m\right)=e\left(h_{ID},S\right)\cdot H_{4}\left(m\right)$ for all i∈[0,n]. It is straightforward that the correctness holds along with the decryption algorithm.

**Perfect consistency**: On input $\left(C_{A},w_{ID_{A}}\right)$ and $\left(C_{B},w_{ID_{B}}\right)$, the filtered equality test algorithm obtains $z_{A}$ by computing

$$\begin{array}{cc} z_{A} & =\frac{C_{A,4}}{\prod_{i=0}^{n}e(C_{A,1,i},w_{ID_{A},i})}=\frac{\prod_{i=0}^{n}e{\left(h_{ID_{A}},S_{i}\right)}^{rh^{i}}\cdot H_{4}\left(m_{A}\right)}{\prod_{i=0}^{n}e(g^{rh^{i}},h_{ID_{A}}^{\left(s_{i}+a_{i}\right)})}\\ & =\frac{e{\left(h_{ID_{A}},g\right)}^{r\sum_{i=0}^{n}s_{i}h^{i}}\cdot H_{4}\left(m_{A}\right)}{e{\left(g,h_{ID_{A}}\right)}^{r\sum_{i=0}^{n}\left(s_{i}h^{i}+a_{i}h^{i}\right)}}=\frac{e{\left(h_{ID_{A}},g\right)}^{r\sum_{i=0}^{n}s_{i}h^{i}}\cdot H_{4}\left(m_{A}\right)}{e{\left(g,h_{ID_{A}}\right)}^{r\sum_{i=0}^{n}s_{i}h^{i}+rf\left(H_{2}\left(m_{A}\right)\right)}}. \end{array}$$

If $m_{A}\in \Omega$, we have $f\left(H_{2}\left(m_{A}\right)\right)=\sum_{i=0}^{n}a_{i}H_{2}{\left(m_{A}\right)}^{i}=0$, therefore $z_{A}=\frac{e{\left(h_{ID_{A}},g\right)}^{r\sum_{i=0}^{n}s_{i}h^{i}}\cdot H_{4}\left(m_{A}\right)}{e{\left(g,h_{ID_{A}}\right)}^{r\sum_{i=0}^{n}s_{i}h^{i}}}=H_{4}\left(m_{A}\right)$. Similarly, if $m_{B}\in \Omega$, we can obtain $z_{B}=H_{4}\left(m_{B}\right)$. If $m_{A}=m_{B}$, then $z_{A}=z_{B}$. The filtered equality test algorithm outputs 1.

**Computational soundness**: For any $m_{A}\in \Omega$ and $m_{B}\in \Omega$, by the inference of consistency, $z_{A}$ and $z_{B}$ will be computed as $z_{A}=H_{4}\left(m_{A}\right)$ and $z_{B}=H_{4}\left(m_{B}\right)$, respectively. If $m_{A}\neq m_{B}$, then $z_{A}\neq z_{B}$, this is because $H_{4}\left(m\right)$ is a collision resistant function. Hence the probability that the filtered equality test algorithm returns 1 is negligible. The computational soundness holds.

## 6. Security Proof

**Figure 5:**
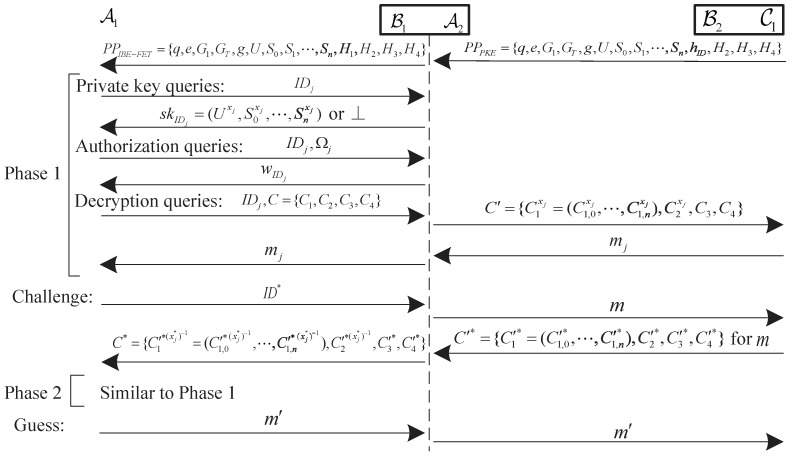
The security proof of IBE-FET.

In this section, based on CBDH assumption, the proposed IBE-FET scheme is proved to be OW-ID-CCA security in the random oracle model. The detail of security proof is shown in Figure 5. Using the same method [32,33,40], we prove the security of the proposed scheme in two steps. We first show that an OW-ID-CCA attack on IBE-FET can be converted to an OW-CCA attack on PKE, then, we show that PKE is OW-CCA secure if the DBDH assumption holds.

**Theorem** **1.** Supposing there is an OW-ID-CCA adversary A that is able to break the proposed scheme with a non-negligible probability ε, then there exists an algorithm B that solves the CBDH problem with the probability at least $\varepsilon^{\prime }=\frac{\varepsilon }{e\left(q_{sk}+q_{aut}+q_{d}+1\right)\left(q_{H_{3}}+1\right)}-\frac{q_{H_{3}}\cdot q_{d}}{2^{l_{1}+l_{2}}\left(q_{H_{3}}+1\right)}$, where $q_{sk}$ is the number of the private key queries, $q_{aut}$ is the number of the authorization queries, $q_{d}$ is the number of the decryption queries and $q_{H_{3}}$ is the number of $H_{3}$ queries, $l_{1}$ is the length of the message and $l_{2}$ is the length of $Z_{q}^{*}$.

**Proof.**  Theorem 1 is proved based on the following Theorem 2 and Theorem 3.  □

To prove Theorem 1, we must convert the OW-ID-CCA attack on an IBE-FET scheme to an OW-CCA attack on a PKE scheme. A related PKE scheme is described below.

- **Setup**: Given a security parameter *k*, the system executes as follows:
  (1) Chooses a bilinear pairing: $e:G_{1}\times G_{1}\to G_{T}$, where $G_{1}$ and $G_{T}$ are two cyclic groups with prime order *q*, *g* is a generator of $G_{1}$.
  (2) Randomly picks $h_{ID}\in G_{1}$, $u,s_{0},s_{1},\cdot \cdot \cdot ,s_{n}\in Z_{q}^{*}$ and computes $U=g^{u}$, $S_{0}=g^{s_{0}}$, $S_{1}=g^{s_{1}}$, ···, $S_{n}=g^{s_{n}}$.
  (3) Chooses three one-way hash functions: $H_{2}:{\left\{0,1\right\}}^{l_{1}}\to Z_{q}^{*}\;$, $H_{3}:G_{T}\to {\left\{0,1\right\}}^{l_{1}+l_{2}}$, $H_{4}:{\left\{0,1\right\}}^{l_{1}}\to G_{T}$, where $l_{1}$ is the length of the message and $l_{2}$ is the length of $Z_{q}^{*}$.
  The system parameters are $PP_{PKE}=\left\{e,G_{1},G_{T},q,g,U,S_{0},S_{1},\cdot \cdot \cdot ,S_{n},h_{ID},H_{2},H_{3},H_{4}\right\}$ and the pravate key are $sk_{ID}=\left\{h_{ID}^{u},h_{ID}^{s_{0}},h_{ID}^{s_{1}},\cdot \cdot \cdot ,h_{ID}^{s_{n}}\right\}$.
- **Encrypt**: Given the message *m*, the sender executes as follows:
  (1) Randomly chooses $r,t\in Z_{q}^{*}$.
  (2) Computes $h=H_{2}\left(m\right)$, $S=\prod_{i=0}^{n}S_{i}^{rh^{i}}$, $C_{1}=\left\{C_{1,0}=g^{r},C_{1,1}=g^{rh},\cdot \cdot \cdot ,C_{1,n}=g^{rh^{n}}\;\right\}$, $C_{2}=g^{t}$, $C_{3}=\left(m||r\right)⊕H_{3}\left(e{\left(h_{ID},U\right)}^{t}\right)$, $C_{4}=e\left(h_{ID},S\right)\cdot H_{4}\left(m\right)$.
  The ciphertext is $C=\{C_{1},C_{2},C_{3},C_{4}\}$, where $C_{1}=\left(C_{1,0},C_{1,1},\cdot \cdot \cdot ,C_{1,n}\right)$.
- **Decrypt**: Given the ciphertexts *C* and the private key $sk_{ID}$, the receiver works as follows:
  (1) Computes $C_{3}⊕H_{3}\left(e\left(h_{ID}^{u},C_{2}\right)\right)=m\left|\right|r$ and $h=H_{2}\left(m\right)$.
  (2) Verifies
    $$C_{1,i}=g^{rh^{i}}\;\mathrm{and}\;C_{4}=\prod_{i=0}^{n}e(h_{ID}^{s_{i}},C_{1,i})\cdot H_{4}\left(m\right)$$
    for all i∈[0,n]. If holds, it outputs *m*. Otherwise, it outputs ⊥.

**Theorem** **2.** *Supposing there is an OW-ID-CCA adversary $A_{1}$ that is able to break the proposed IBE-FET scheme with a non-negligible probability $\varepsilon_{1}$, then there exists an OW-CCA adversary $B_{1}$ that can break the PKE scheme with the probability at least $\varepsilon_{1}^{\prime }=\frac{\varepsilon_{1}}{e(q_{sk}+q_{aut}+q_{d}+1)}$, where $q_{sk}$ is the number of the private key queries, $q_{aut}$ is the number of the authorization queries and $q_{d}$ is the number of the decryption queries.*

**Proof.**  In order to convert an OW-ID-CCA attack on IBE-FET to an OW-CCA attack on PKE, we can construct a simulator $C_{1}$ to execute the game between $A_{1}$ and $B_{1}$.  □

**Initialization**: $C_{1}$ runs the Setup algorithm of PKE and returns the system parameters $PP_{PKE}=\left\{q,e,G_{1},G_{T},g,U,S_{0},S_{1},\cdot \cdot \cdot ,S_{n},h_{ID},H_{2},H_{3},H_{4}\right\}$ to $B_{1}$. $A_{1}$ interacts with $B_{1}$ as follows.

**Setup**: $B_{1}$ chooses a hash function $H_{1}$ and returns the system parameters $PP_{IBE-FET}=\left\{q,e,G_{1},G_{T},g,U,S_{0},S_{1},\cdot \cdot \cdot ,S_{n},H_{1},H_{2},H_{3},H_{4}\right\}$ to $A_{1}$. For the quickly respond and consistency, $B_{1}$ maintains an initially empty list $H_{1}^{list}$ of tuples $\left(ID_{j},h_{1,j},x_{j},c_{j}\right)$.

**Phase 1**: $A_{1}$ makes the following queries.

- **Hash $H_{1}$ queries**: $A_{1}$ submits a query on $ID_{j}$, $B_{1}$ checks the list $H_{1}^{list}$ and performs as below:
  - If $H_{1}^{list}$ contains $\left(ID_{j},h_{1,j},x_{j},c_{j}\right)$, $B_{1}$ responds with previous value $h_{1,j}$ to $A_{1}$.
  - If $H_{1}^{list}$ doesn’t contain $\left(ID_{j},h_{1,j},x_{j},c_{j}\right)$, based on the Coron’s technology [50], $B_{1}$ tosses a coin $c_{j}\in \left\{0,\;\;1\right\}$ that yield 0 with probability δ and 1 with probability 1−δ. $B_{1}$ randomly chooses $x_{j}\in Z_{q}^{*}$. If $c_{j}=0$, $B_{1}$ computes $h_{1,j}=g^{x_{j}}$. If $c_{j}=1$, $B_{1}$ computes $h_{1,j}=h_{ID}^{x_{j}}$. Finally, $B_{1}$ adds the tuple $\left(ID_{j},h_{1,j},x_{j},c_{j}\right)$ to the list $H_{1}^{list}$ and returns $h_{1,j}$ to $A_{1}$.
- **Private key queries**: $A_{1}$ submits a private key query on $ID_{j}$, $B_{1}$ makes the hash $H_{1}$ query on $ID_{j}$ to obtain the corresponding tuple $\left(ID_{j},h_{1,j},x_{j},c_{j}\right)$.
  - If $c_{j}=0$, $B_{1}$ returns $sk_{ID_{j}}=\left\{U^{x_{j}},S_{0}^{x_{j}},S_{1}^{x_{j}},\cdot \cdot \cdot ,S_{n}^{x_{j}}\right\}$ to $A_{1}$.
  - If $c_{j}=1$, $B_{1}$ returns ⊥.
- **Decryption queries**: $A_{1}$ submits a decryption query on $ID_{j}$ and $C=\{C_{1},C_{2},C_{3},C_{4}\}$, $B_{1}$ makes the hash $H_{1}$ query on $ID_{j}$ to obtain the corresponding tuple $\left(ID_{j},h_{1,j},x_{j},c_{j}\right)$.
  - If $c_{j}=0$, $B_{1}$ obtains $sk_{ID_{j}}=\left\{U^{x_{j}},S_{0}^{x_{j}},\cdot \cdot \cdot ,S_{n}^{x_{j}}\right\}$ and decrypts *C* using $sk_{ID_{j}}$.
  - If $c_{j}=1$, $B_{1}$ obtains $h_{1,j}=h_{ID}^{x_{j}}$ and computes $sk_{ID_{j}}=\left\{{\left(h_{ID}^{x_{j}}\right)}^{u},{\left(h_{ID}^{x_{j}}\right)}^{s_{0}},{\left(h_{ID}^{x_{j}}\right)}^{s_{1}},\cdot \cdot \cdot ,{\left(h_{ID}^{x_{j}}\right)}^{s_{n}}\right\}$. Then $B_{1}$ sets $C^{\prime }=\left\{C_{1}^{x_{j}}=\left(C_{1,0}^{x_{j}},C_{1,1}^{x_{j}},\cdot \cdot \cdot ,C_{1,n}^{x_{j}}\right)\;,C_{2}^{x_{j}},C_{3},C_{4}\right\}$. Note that the IBE-FET decryption of *C* using $sk_{ID_{j}}=\left\{{\left(h_{ID}^{x_{j}}\right)}^{u},{\left(h_{ID}^{x_{j}}\right)}^{s_{0}},{\left(h_{ID}^{x_{j}}\right)}^{s_{1}},\cdot \cdot \cdot ,{\left(h_{ID}^{x_{j}}\right)}^{s_{n}}\right\}$ is the same as the PKE decryption of $C^{\prime }$ using $sk_{ID_{j}}=\left\{h_{ID}^{u},h_{ID}^{s_{0}},h_{ID}^{s_{1}},\cdot \cdot \cdot ,h_{ID}^{s_{n}}\right\}$ because $e\left({\left(h_{ID}^{x_{j}}\right)}^{u},C_{2}\right)=e\left(h_{ID}^{u},C_{2}^{x_{j}}\right)$ and $e\left({\left(h_{ID}^{x_{j}}\right)}^{s_{i}},C_{1,i}\right)=e\left(h_{ID}^{s_{i}},C_{1,i}^{x_{j}}\right)$ for any i∈[0,n]. $B_{1}$ makes the decryption query on $C^{\prime }$ to $C_{1}$ and returns the response of $C_{1}$ to $A_{1}$.
- **Authorization queries**: $A_{1}$ submits an authorization query on $ID_{j}$ and the message set $\Omega_{j}$, $B_{1}$ makes the private key query on $ID_{j}$ to obtain $sk_{ID_{j}}$. Then $B_{1}$ runs the authorization algorithm and returns the warrant $w_{ID_{j}}$ to $A_{1}$.

**Challenge**: $A_{1}$ chooses the challenge identity $ID^{*}$ and returns it to $B_{1}$. Here, $ID^{*}$ does not appear in the private key queries of Phase 1. Then $B_{1}$ makes the hash $H_{1}$ query on $ID^{*}$ to get the tuple $\left(ID^{*},h_{1,j}^{*},x_{j}^{*},c_{j}^{*}\right)$ and executes as follows:

- If $c_{j}^{*}=0$, $B_{1}$ returns ⊥.
- If $c_{j}^{*}=1$, $C_{1}$ randomly chooses $m^{*}$ and returns a PKE challenge ciphertext $C^{\prime *}=\left\{C_{1}^{\prime *}=\left(C_{1,0}^{\prime *},C_{1,1}^{\prime *},\cdot \cdot \cdot ,C_{1,n}^{\prime *}\right),C_{2}^{\prime *},C_{3}^{\prime *},C_{4}^{\prime *}\right\}$ on $m^{*}$ to $B_{1}$. Then $B_{1}$ returns $C^{*}=\left\{{C_{1}^{\prime *}}^{{\left(x_{j}^{*}\right)}^{-1}}=\left({C_{1,0}^{\prime *}}^{{\left(x_{j}^{*}\right)}^{-1}},{C_{1,1}^{\prime *}}^{{\left(x_{j}^{*}\right)}^{-1}}\cdot \cdot \cdot ,{C_{1,n}^{\prime *}}^{{\left(x_{j}^{*}\right)}^{-1}}\right),{C_{2}^{\prime *}}^{{\left(x_{j}^{*}\right)}^{-1}},C_{3}^{\prime *},C_{4}^{\prime *}\right\}$ to $A_{1}$.

**Phase 2**: $A_{1}$ makes queries as done in Phase 1.

- **Private key queries**: If $ID^{*}\neq ID_{j}$, $B_{1}$ responds as in Phase 1. Otherwise, $B_{1}$ returns ⊥.
- **Decryption queries**: If $\left(ID^{*},C^{*}\right)\neq \left(ID_{j},C_{j}\right)$, $B_{1}$ responds as in Phase 1. Otherwise, $B_{1}$ returns ⊥.
- **Authorization queries**: $B_{1}$ responds as in Phase 1.

**Guess**: $A_{1}$ outputs a guess $m^{\prime }$ for $m^{*}$. $B_{1}$ outputs a guess $m^{\prime }$ for $m^{*}$.

We define the following three events:

- $\zeta_{1}$ : $B_{1}$ aborts in the private key query during Phase 1 or Phase 2.
- $\zeta_{2}$ : $B_{1}$ aborts in the challenge phase.
- $\zeta_{3}$ : $B_{1}$ aborts in the decryption query in Phase 2.

Thus, we have

$$\mathrm{Pr}\left[\neg \zeta_{1}\wedge \neg \zeta_{2}\wedge \neg \zeta_{3}\right]⩾\left(1-\delta \right)\delta^{\left(q_{sk}+q_{aut}+q_{d}\right)}.$$

Clearly, $\left(1-\delta \right)\delta^{\left(q_{sk}+q_{aut}+q_{d}\right)}$ can obtain the maximized when $\delta =1-\frac{1}{\left(q_{sk}+q_{aut}+q_{d}+1\right)}$. The probability that $B_{1}$ does not abort is at least $\frac{1}{\left(q_{sk}+q_{aut}+q_{d}+1\right)}$. Therefore, the advantage of $B_{1}$ is at least $\frac{\varepsilon_{1}}{e(q_{sk}+q_{aut}+q_{d}+1)}$.

**Theorem** **3.** Supposing there is an OW-CCA adversary $A_{2}$ that is able to break the PKE scheme with a non-negligible probability $\varepsilon_{2}$, then there exists an algorithm $B_{2}$ that solves the CBDH problem with the probability at least $\varepsilon_{2}^{\prime }=\frac{\varepsilon_{2}}{q_{H_{3}}+1}-\frac{q_{H_{3}}\cdot q_{d}}{\left(q_{H_{3}}+1\right)\cdot 2^{l_{1}+l_{2}}}$, where $q_{H_{3}}$ is the number of $H_{3}$ queries and $q_{d}$ is the number of the decryption queries, $l_{1}$ is the length of the message and $l_{2}$ is the length of $Z_{q}^{*}$.

**Proof.**  Let $\varepsilon_{2}=Adv_{PKE,\;A_{2}}^{OW-CCA}$ represent the advantage of $A_{2}$ in the OW-CCA security game. According to schemes [23,24,25,26,27,28,29,30,31], this theorem is proved by performing a series of games. Let $Q_{i}$ denote the event that $m^{\prime }=m^{*}$ in Game *i* (i=0,1,2). We define the Game 0 to be the real security game against the adversary in Definition 2. Then, we can modify the last game in an indistinguishable way to obtain the next game. The adversary has no advantage unconditionally in last game, thus he can make the queries many times, then the event will happen in the next game. Since each game is indistinguishable from the next, to prove the real security game, we can show that the probability of an event is negligible if the DBDH assumption holds. The detailed process is shown as follows.  □

**Game 0**:

**1. Initial phase**: $B_{2}$ generates $u,s_{0},s_{1},\cdot \cdot \cdot ,s_{n}\in Z_{q}^{*}$ and $h_{ID}\in G_{1}$ by running the Setup algorithm, then computes $U=g^{u},\;\;\;S_{0}=g^{s_{0}},S_{1}=g^{s_{1}},\cdot \cdot \cdot ,S_{n}=g^{s_{n}}$. Finally, $B_{2}$ returns the system parameters $PP_{PKE}=\left\{q,e,G_{1},G_{T},g,U,S_{0},S_{1},\cdot \cdot \cdot ,S_{n},h_{ID},H_{2},H_{3},H_{4}\right\}$ to $A_{2}$. For the quickly respond and consistency, $B_{2}$ maintains an initially empty list $H_{3}^{list}$ of tuples $\left(\Phi_{i},h_{3,i}\right)$.

**2. Query phase**: $B_{2}$ works as follows:

- **Hash $H_{3}$ queries**: $A_{2}$ makes a hash $H_{3}$ query on $\Phi_{i}$, $B_{2}$ checks the list $H_{3}^{list}$ and performs as follows.
  - If $H_{3}^{list}$ includes $\left(\Phi_{i},h_{3,i}\right)$, $B_{2}$ returns $h_{3,i}$ to $A_{2}$.
  - If $H_{3}^{list}$ doesn’t include $\left(\Phi_{i},h_{3,i}\right)$, $B_{2}$ selects a random sting $h_{3,i}\in {\left\{0,1\right\}}^{l_{1}+l_{2}}$ and returns $h_{3,i}$ to $A_{2}$.
- **Decryption queries**: $A_{2}$ makes a decryption query on *C*, $B_{2}$ returns *m* to $A_{2}$ by running the decryption algorithm using the private key.

**3. Challenge phase**: For any $m^{*}$, $B_{2}$ randomly chooses $r,t\in Z_{q}^{*}$ and computes $h=H_{2}\left(m^{*}\right)$, $S=\prod_{i=0}^{n}S_{i}^{rh^{i}}$ and defines the challenge ciphertexts

$$C^{*}=\left\{C_{1}^{*}=\left(C_{1,0}^{*},C_{1,1}^{*},\cdot \cdot \cdot ,C_{1,n}^{*}\right),C_{2}^{*},C_{3}^{*},C_{4}^{*}\right\}$$

as follows:

$C_{1}^{*}=\left\{C_{1,0}^{*}=g^{r},C_{1,1}^{*}=g^{rh},\cdot \cdot \cdot ,C_{1,n}^{*}=g^{rh^{n}}\right\}$,

$C_{2}^{*}=g^{t}$,

$C_{3}^{*}=(m^{*}\left||r\right)⊕H_{3}\left(e{\left(h_{ID},U\right)}^{t}\right)$,

$C_{4}^{*}=e\left(h_{ID},S\right)\cdot H_{4}\left(m^{*}\right)$.

**4. Output phase**: $A_{2}$ outputs a guess $m^{\prime }$ for $m^{*}$.

Thus, the advantage of $A_{2}$ winning in Game 0 is

(1) $$\begin{array}{c} Adv_{PKE,\;A_{2}}^{OW-CCA}=Pr\left[Q_{0}\right]. \end{array}$$

**Game 1**:

**1. Initial phase**: $B_{2}$ responds as in Game 0.

**2. Query phase**: $B_{2}$ works as follows:

- **Hash $H_{3}$ queries**: $A_{2}$ makes a hash $H_{3}$ query on $\Phi_{i}$, $B_{2}$ checks the list $H_{3}^{list}$ and performs as follows.
  - If $H_{3}^{list}$ includes $\left(\Phi_{i},h_{3,i}\right)$. When $\Phi_{i}=e{\left(h_{ID},U\right)}^{t}$, $B_{2}$ defines $\omega_{1}^{*}=H_{3}\left(e{\left(h_{ID},U\right)}^{t}\right)$ as $h_{3,i}$ and returns $\omega_{\;1}^{*}$ to $A_{2}$; otherwise, $B_{2}$ returns $h_{3,i}$ to $A_{2}$.
  - If $H_{3}^{list}$ doesn’t include $\left(\Phi_{i},h_{3,i}\right)$, $B_{2}$ selects a random sting $h_{3,i}\in {\left\{0,1\right\}}^{l_{1}+l_{2}}$ and returns $h_{3,i}$ to $A_{2}$.
- **Decryption queries**: $B_{2}$ responds a decryption query as in Game 0.

**3. Challenge phase**: For any $m^{*}$, $B_{2}$ randomly chooses $r,t\in Z_{q}^{*}$, $\omega_{1}^{*}\in {\left\{0,1\right\}}^{l_{1}+l_{2}}$ and computes $h=H_{2}\left(m^{*}\right)$, $S=\prod_{i=0}^{n}S_{i}^{{}^{rh^{i}}}$ and defines the challenge ciphertexts

$$C^{*}=\left\{C_{1}^{*}=\left(C_{1,0}^{*},C_{1,1}^{*},\cdot \cdot \cdot ,C_{1,n}^{*}\right),C_{2}^{*},C_{3}^{*},C_{4}^{*}\right\}$$

as follows:

$C_{1}^{*}=\left\{C_{1,0}^{*}=g^{r},C_{1,1}^{*}=g^{rh},\cdot \cdot \cdot ,C_{1,n}^{*}=g^{rh^{n}}\;\right\}$,

$C_{2}^{*}=g^{t}$,

$C_{3}^{*}=(m^{*}\left||r\right)⊕\omega_{1}^{*}$,

$C_{4}^{*}=e\left(h_{ID},S\right)\cdot H_{4}\left(m^{*}\right)$.

**4. Update phase**: $B_{2}$ adds the tuple $\left(e{\left(h_{ID},U\right)}^{t},\omega_{1}^{*}\right)$ to the list $H_{3}^{list}$.

**5. Output phase**: $A_{2}$ outputs a guess $m^{\prime }$ for $m^{*}$.

Compared to Game 0, the value of $H_{3}$ is replaced by a random value $\omega_{1}^{*}$ in Game 1. According to the random oracle model, the advantage of $A_{2}$ winning in Game 1 is identical to Game 0. Thus

(2) $$\begin{array}{c} Adv_{PKE,\;A_{2}}^{OW-CCA}=Pr\left[Q_{0}\right]=Pr\left[Q_{1}\right]. \end{array}$$

**Game 2**:

**1. Initial phase**: $B_{2}$ responds as in Game 1.

**2. Query phase**: $B_{2}$ works as follows:

- **Hash $H_{3}$ queries**: $A_{2}$ makes a hash $H_{3}$ query on $\Phi_{i}$, $B_{2}$ checks the list $H_{3}^{list}$ and performs as follows.
  - If $H_{3}^{list}$ includes $\left(\Phi_{i},h_{3,i}\right)$. When $\Phi_{i}=e{\left(h_{ID},U\right)}^{t}$, $B_{2}$ returns ⊥. Define this event as $E_{1}$; otherwise, $B_{2}$ returns $h_{3,i}$ to $A_{2}$.
  - If $H_{3}^{list}$ does not include $\left(\Phi_{i},h_{3,i}\right)$, $B_{2}$ selects a random sting $h_{3,i}\in {\left\{0,1\right\}}^{l_{1}+l_{2}}$ and returns $h_{3,i}$ to $A_{2}$.
- **Decryption queries**: $A_{2}$ makes a decryption query on *C*. If *C* is equal to the challenge ciphertext $C^{*}$ except $C_{3}$, $B_{2}$ returns ⊥. Otherwise, $B_{2}$ responds as in Game 1.

**3. Challenge phase**: For any $m^{*}$, $B_{2}$ randomly chooses $r,t\in Z_{q}^{*}$, $\omega_{2}^{*}\in {\left\{0,1\right\}}^{l_{1}+l_{2}}$ and computes $h=H_{2}\left(m^{*}\right)$, $S=\prod_{i=0}^{n}S_{i}^{rh^{i}}$ and defines the challenge ciphertexts

$$C^{*}=\left\{C_{1}^{*}=\left(C_{1,0}^{*},C_{1,1}^{*},\cdot \cdot \cdot ,C_{1,n}^{*}\right),C_{2}^{*},C_{3}^{*},C_{4}^{*}\right\}$$

as follows:

$C_{1}^{*}=\left\{C_{1,0}^{*}=g^{r},C_{1,1}^{*}=g^{rh},\cdot \cdot \cdot ,C_{1,n}^{*}=g^{rh^{n}}\right\}$,

$C_{2}^{*}=g^{t}$,

$C_{3}^{*}=\omega_{2}^{*}$,

$C_{4}^{*}=e\left(h_{ID},S\right)\cdot H_{4}\left(m^{*}\right)$.

**4. Update phase**: $B_{2}$ adds the tuple $(e{\left(h_{ID},U\right)}^{t},\omega_{2}^{*}⊕(m^{*}\left||r)\right)$ to the list $H_{3}^{list}$.

**5. Output phase**: $A_{2}$ outputs a guess $m^{\prime }$ for $m^{*}$.

Compared to Game 1, the value of $C_{3}^{*}$ is replaced by a random value $\omega_{2}^{*}$ in Game 2. According to the random oracle model, if the event $E_{1}$ does not occur, Game 2 is the same as Game 1. Therefore

(3) $$\begin{array}{c} |\mathrm{Pr}\left[Q_{2}\right]|-|\mathrm{Pr}\left[Q_{1}\right]|⩽\mathrm{Pr}\left[E_{1}\right]. \end{array}$$

Now, we proof the event $E_{1}$ occurs with negligible probability

(4) $$\begin{array}{c} \mathrm{Pr}\left[E_{1}\right]⩽Adv_{P_{1}}^{CBDH}\cdot q_{H_{3}}+\frac{q_{d}\cdot q_{H_{3}}}{2^{l_{1}+l_{2}}}. \end{array}$$

**Claim** **1.** *Event $E_{1}$ occurs with negligible probability $\mathrm{Pr}[E_{1}]$ in Game 2 if the CBDH problem is intractable.*

**Proof.**  Assume the event $E_{1}$ occurs in Game 2 with a non-negligible probability $\mathrm{Pr}[E_{1}]$, we can construct an algorithm $P_{1}$ that can compute $e{\left(g,g\right)}^{xyz}$ with a non-negligible probability when receiving a random CBDH problem instance $\left(g,g^{x},g^{y},g^{z}\right)$.  □

$P_{1}$ randomly selects $r,s_{0},s_{1},\cdot \cdot \cdot ,s_{n}\in Z_{q}^{*}$, $m^{*}\in \Delta$, $\nu_{1}^{*}\in {\left\{0,1\right\}}^{l_{1}+l_{2}}$ and computes $h=H_{2}\left(m^{*}\right)$. The system parameters are {$h_{ID}=g^{x},U=g^{y},S_{0}=g^{s_{0}},\;\;S_{1}=g^{s_{1}},\cdot \cdot \cdot ,S_{n}=g^{s_{n}},S=\prod_{i=0}^{n}S_{i}^{rh^{i}}=g^{r\sum_{i=0}^{n}s_{i}h^{i}}$}. Then, $P_{1}$ calculates $C_{1}^{*}=\left\{C_{1,0}^{*}=g^{r},C_{1,1}^{*}=g^{rh},\cdot \cdot \cdot ,C_{1,n}^{*}=g^{rh^{n}}\right\}$, $C_{2}^{*}=g^{z}$, $C_{3}^{*}=\nu_{1}^{*}$ and $C_{4}^{*}=e\left(h_{ID},S\right)\cdot H_{4}\left(m^{*}\right)$ as the challenge ciphertexts and adds $(⊥\;\;,\nu_{1}^{*}⊕(m^{*}\left||r)\right)$ into the list $H_{3}^{list}$. Finally, $P_{1}$ returns $PP_{PKE}=\left\{q,e,G_{1},G_{T},g,U,S_{0},S_{1},\cdot \cdot \cdot ,S_{n},S,h_{ID},H_{2},H_{3},H_{4}\right\}$ and the challenge ciphertexts $C^{*}=\left\{C_{1}^{*},C_{2}^{*},C_{3}^{*},C_{4}^{*}\right\}$ to $A_{2}$. $A_{2}$ makes the following queries:

- **Hash $H_{3}$ queries**: $P_{1}$ responds as in Game 2.
- **Decryption queries**: $A_{2}$ makes a decryption query on *C*. If $C_{1}=C_{1}^{*}$, $C_{2}=C_{2}^{*}$, $C_{3}\neq C_{3}^{*}$, $C_{4}=C_{4}^{*}$, $P_{1}$ returns ⊥. Otherwise, $P_{1}$ searches the list $H_{3}^{list}$ to get $h_{3,i}$ and computes $m^{*}\left|\right|r=h_{3,i}⊕C_{3}^{*}$, $h=H_{2}\left(m^{*}\right)$. If $C_{1,i}^{*}=g^{rh^{i}}$ and $C_{4}^{*}=\prod_{i=0}^{n}e{\left(h_{ID},C_{1,i}^{*}\right)}^{s_{i}}\cdot H_{4}\left(m^{*}\right)$ are hold for all i∈[0,n], $P_{1}$ returns $m^{*}$ to $A_{2}$.

If the following two cases holds, $P_{1}$ can solve the CBDH problem:

1. $A_{2}$ has never made a hash $H_{3}$ query on $e{\left(h_{ID},C_{2}\right)}^{y}$ before a decryption query on $C=\{C_{1},C_{2},C_{3},C_{4}\}$. In this case, $P_{1}$ returns ⊥. If *C* is a valid ciphertext, it means $A_{2}$ guesses the value of $h_{3,i}$ correctly. Thus the probability is $\frac{1}{2^{l_{1}+l_{2}}}$.
2. The event $E_{1}$ occurs in the hash $H_{3}$ queries. It means that the list $H_{3}^{list}$ includes the tuple $\left(e{\left(h_{ID},C_{2}\right)}^{y},\;⊥\right)$. The probability is $\frac{\mathrm{Pr}[E_{1}]}{q_{H_{3}}}$.

Let $X_{1}$ to be event that the ciphertext is valid when $P_{1}$ returns ⊥ in the case 1. Then we have

(5) $$\begin{array}{c} \mathrm{Pr}\left[X_{1}\right]⩽\frac{q_{d}}{2^{l_{1}+l_{2}}}. \end{array}$$

Let $X_{2}$ to be event in case 2 that $P_{1}$ obtains $e{\left(g,g\right)}^{xyz}$ as a solution of the CBDH problem. If $X_{1}$ does not occur and $\left(e{\left(h_{ID},C_{2}\right)}^{y},\;⊥\right)$ appears in the list $H_{3}^{list}$ with the probability at least $\mathrm{Pr}[E_{1}]$. So

(6) $$\begin{array}{c} \mathrm{Pr}\left[X_{2}\left|\neg \;\;X_{1}\right.\right]=\frac{\mathrm{Pr}[E_{1}]}{q_{H_{3}}}. \end{array}$$

Then

$$\begin{array}{cc} \mathrm{Pr}[X_{2}] & =\mathrm{Pr}\left[X_{2}\left|X_{1}\right.\right]\mathrm{Pr}\left[X_{1}\right]+\mathrm{Pr}\left[X_{2}\left|\neg X_{1}\right.\right]\mathrm{Pr}\left[\neg X_{1}\right]\\ & ⩾\mathrm{Pr}\left[X_{2}\left|\neg X_{1}\right.\right]\mathrm{Pr}\left[\neg X_{1}\right]\\ & =\mathrm{Pr}\left[X_{2}\left|\neg X_{1}\right.\right]\left(1-\mathrm{Pr}\left[X_{1}\right]\right)\\ & =\mathrm{Pr}\left[X_{2}\left|\neg X_{1}\right.\right]-\mathrm{Pr}\left[X_{2}\left|\neg X_{1}\right.\right]\mathrm{Pr}\left[X_{1}\right]\\ & ⩾\mathrm{Pr}\left[X_{2}\left|\neg X_{1}\right.\right]-\mathrm{Pr}\left[X_{1}\right]\\ & =\frac{\mathrm{Pr}[X_{1}]}{q_{H_{3}}}-\frac{q_{d}}{2^{l_{1}+l_{2}}}. \end{array}$$

So, we obtain

(7) $$\begin{array}{c} Adv_{P_{1}}^{CBDH}⩾\frac{\mathrm{Pr}[E_{1}]}{q_{H_{3}}}-\frac{q_{d}}{2^{l_{1}+l_{2}}}. \end{array}$$

According to the assumption, if $\mathrm{Pr}[E_{1}]$ is non-negligible, the advantage $Adv_{P_{1}}^{CBDH}$ is non-negligible. The proof of Claim 1 is completed.

**Claim** **2.** *Event $Q_{2}$ occurs with negligible probability $\mathrm{Pr}[Q_{2}]$ in Game 2 if the CBDH problem is intractable.*

**Proof.**  Assume the event $Q_{2}$ occurs in Game 2 with a non-negligible probability $\mathrm{Pr}[Q_{2}]$, we can construct an algorithm $P_{2}$ that can compute $e{\left(g,g\right)}^{xyz}$ with a non-negligible probability when receiving a random CBDH problem instance $\left(g,g^{x},g^{y},g^{z}\right)$.  □

$P_{2}$ randomly selects $t,s_{1},s_{2},\cdot \cdot \cdot ,s_{n}\in Z_{q}^{*}$, $\nu_{1}^{*}\in {\left\{0,1\right\}}^{l_{1}+l_{2}}$, $\nu_{2}^{*}\in G_{T}$, $m^{*}\in \Delta$ and computes $h=H_{2}\left(m^{*}\right)$. The system parameters are {$h_{ID}=g^{x},S_{0}=g^{y},S_{1}=g^{s_{1}},S_{2}=g^{s_{2}},\cdot \cdot \cdot ,S_{n}=g^{s_{n}}$}. Then, $P_{2}$ calculates $C_{1}^{*}=\{C_{1,0}^{*}=g^{z},C_{1,1}^{*}=g^{zh},C_{1,2}^{*}=g^{zh^{2}},\cdot \cdot \cdot ,C_{1,n}^{*}=g^{zh^{n}}$, $C_{2}^{*}=g^{t}$, $C_{3}^{*}=\nu_{1}^{*}$ and $C_{4}^{*}=\nu_{2}^{*}\cdot H_{4}\left(m^{*}\right)$ as the challenge ciphertexts and adds $(⊥,\nu_{1}^{*}⊕(m^{*}\left||r)\right)$ into the list $H_{3}^{list}$. And $P_{2}$ returns $PP_{PKE}=\left\{q,e,G_{1},G_{T},g,U,S_{0},S_{1},S_{2},\cdot \cdot \cdot ,S_{n},h_{ID},H_{2},H_{3},H_{4}\right\}$ and the challenge ciphertexts $C^{*}=\left\{C_{1}^{*},C_{2}^{*},C_{3}^{*},C_{4}^{*}\right\}$ to $A_{2}$.

$A_{2}$ interacts with $P_{2}$ as Game 2.

Finally, $P_{2}$ obtains $e{\left(g,g\right)}^{xyz}$ by computing

$$e{\left(h_{ID},C_{1,0}^{*}\right)}^{y}=\frac{C_{4}^{*}}{H_{4}\left(m^{*}\right)\cdot \prod_{i=1}^{n}e{\left(h_{ID},C_{1,i}^{*}\right)}^{s_{i}}}.$$

Therefore, we have

(8) $$\begin{array}{c} \mathrm{Pr}\left[Q_{2}\right]⩽Adv_{P_{2}}^{CBDH}. \end{array}$$

According to the assumption, if $\mathrm{Pr}[Q_{2}]$ is non-negligible, the advantage $Adv_{P_{2}}^{CBDH}$ is non-negligible. The proof of Claim 2 is completed.

Owing to the Equations (1)–(8), we can claim that

$$\begin{array}{cc} Adv_{PKE,\;A_{2}}^{OW-CCA} & =\mathrm{Pr}[Q_{0}]\;\;\\ & =\mathrm{Pr}[Q_{1}]\\ & ⩽\mathrm{Pr}\left[Q_{2}\right]+Adv^{CBDH}\cdot q_{H_{3}}+\frac{q_{H_{3}}\cdot q_{d}}{2^{l_{1}+l_{2}}}\;\\ & ⩽\left(q_{H_{3}}+1\right)\cdot Adv^{CBDH}+\frac{q_{H_{3}}\cdot q_{d}}{2^{l_{1}+l_{2}}}. \end{array}$$

So, Theorem 3 has been proved.

According to Theorem 2 and Theorem 3, we can show that the proposed IBE-FET scheme satisfies OW-ID-CCA security. Assume an OW-ID-CCA adversary A is able to against IBE-FET with the probability ε, then there the algorithm B can solve the CBDH problem with the probability at least $\varepsilon^{\prime }=\frac{\varepsilon }{e\left(q_{dk}+q_{Aut}+q_{d}+1\right)\left(q_{H_{3}}+1\right)}-\frac{q_{H_{3}}\cdot q_{d}}{\left(2^{l_{1}+l_{2}}\right)\left(q_{H_{3}}+1\right)}.$

## 7. Comparison and Performance Evaluation

In this section, we present the comparisons between the proposed IBE-FET scheme and the existing related schemes [23,24,25,30,32,33,39].

### 7.1. Comparison

The comparison for the proposed IBE-FET scheme and the related schemes [23,24,25,30,32,33,39] is given in Table 1. Let ET be the quality test, FET be the filtered quality test, ID be the identity-based and ROM be the random oracle model. Let ✓ denote “satisfy” and ✗ denote “not satisfy”.

From Table 1, it is clearly observed that scheme [39] and the proposed scheme support the filtered equality test while other schemes only provide the equality test. Schemes [32,33] and the proposed scheme adopt the identity-based cryptography which can avoid the certificate management problem, while other schemes adopt public key cryptography. With regard to security, all schemes are provably secure based on basic assumptions in the random oracle except scheme [39]. However, none of the schemes [23,24,25,30,32,33,39] could satisfy both the properties of the filtered equality test and of the identity-based one, only our scheme can do it.

### 7.2. Computation Cost

For computation complexity estimation, the time cost for performing the cryptographic operations is defined as follows. Let $T_{E}$ and $T_{P}$ denote the time of a scale multiplication operation and a bilinear pairing operation, respectively. The time of a map-to-point hash function operation is denoted as $T_{H}$. Other lightweight operations (point addition, one way hash function operation) are not taken into account.

To offer the security level of 80-bit, we adopt the symmetric bilinear pairing $e:G_{1}\times G_{1}\to G_{T}$, here $G_{1}$ is the cyclic group generated by a generator *g* with the order *q* on a super singular elliptic curve $E:y^{2}=x^{3}+x\;\mathrm{mod}\;p$ with embedding degree 2. *p* is 512-bit prime number and *q* is 160-bit Solinas prime number, which satisfy q·12·r=p+1. Using the MIRACL Crypto SDK [51], the running time of the cryptographic operations are quantified. The experiment is run on an Intel Core i5-4590, 3.3GHz CPU, 8 gigabytes memory with Windows 7 environment. Table 2 lists the average execution times of cryptographic operations $T_{E}$, $T_{P}$, and $T_{H}$.

Based on the experimental results, the computation cost of the proposed IBE-FET scheme and the related schemes [23,24,25,30,32,33,39] are summarized in Table 3.

In the encryption phase, the proposed scheme needs to execute n+3 scalar multiplication operations, two bilinear pairing operations and two map-to-point hash operations; therefore, the total encryption time is $\left(n+3\right)T_{E}+2T_{P}+2T_{H}=3.7770n+48.8996$ ms. In the decryption phase, the proposed scheme needs to execute n+1 scalar multiplication operations, n+2 bilinear pairing operations and one map-to-point hash operation; therefore, the total decryption time is $\left(n+1\right)T_{E}+\left(n+2\right)T_{P}+1T_{H}=12.8561n+31.6404$ ms. In the authorization phase, the proposed scheme needs to execute n+1 scalar multiplication operations; therefore, the total authorization time is $\left(n+1\right)T_{E}=3.7770n+3.7770$ ms. In the test phase, the proposed scheme needs to execute n+1 bilinear pairing operations; therefore, the total test time is $\left(n+1\right)T_{P}=9.0791n+9.0791$ ms. From Table 3, we can arrive at the fact that the computational cost of the proposed scheme is higher than those of other schemes [23,24,25,30,32,33,39] in both encryption and decryption phases. In terms of authorization and test phases, the proposed scheme has the same computational cost as scheme [39], which is more than those of other schemes [23,24,25,30,32,33,39].

**Figure 6:**
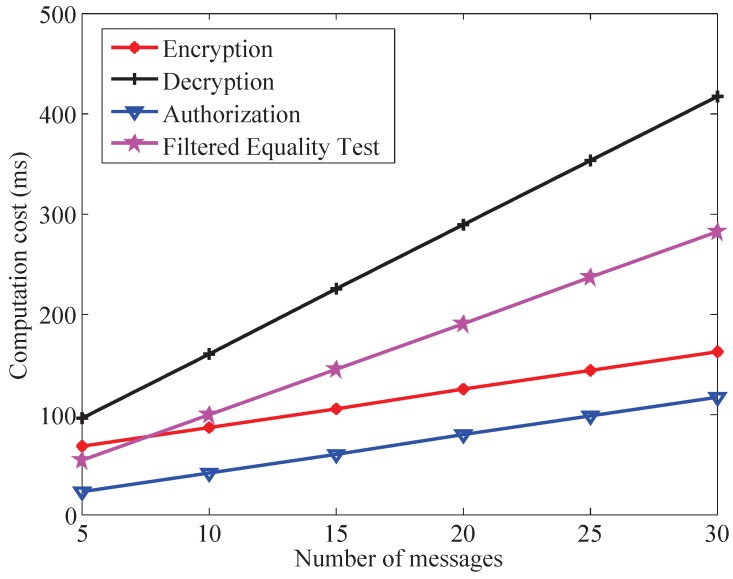
Computational cost with different number of messages.

Figure 6 describes the relationship between the computational cost of the proposed scheme and the number of message *n*. As shown in Figure 6, the total computational cost increases linearly with the number of message in all phases. The computational cost is equal to 67.7496, 95.9209, 22.6270 and 54.4746 ms when n=5, that is equal to 162.2096, 417.3234, 117.0870 and 281.4521 ms when n=30, in encryption, decryption, authorization, and equation test phase of the proposed scheme, respectively. Based on the above analysis, the computational cost of the proposed scheme is feasible.

### 7.3. Communication Cost

We compare the communication cost of the proposed IBE-FET and those of the related schemes [23,24,25,30,32,33,39] in this section. The communication cost is represented by the size of message transmitted. The sender transmits the ciphertext to the cloud server for storing and a warrant is transmitted from the receiver to the cloud server in order to perform the filter equality test. Therefore, the communication cost is generated as a result of the communication between the sender and the cloud server and between the receiver and the cloud server. Let |PK|, |CT|, |WT| denote the sizes of the public key, ciphertext and warrant, respectively. Let $|G_{1}|$ be the length of the element in group $G_{1}$, $|G_{T}|$ be the length of the element in group $G_{T}$, $|Z_{q}|$ be the element’s length of $Z_{q}$. Since the size of *q* is 512 bits (64 bytes), therefore the sizes of the elements in group $G_{1}$ and $G_{T}$ are 512 bits (64 bytes) and 3072 bits (384 bytes) respectively. The length of $Z_{q}$ is 512 bits (64 bytes). Based on the above analysis, in the proposed scheme, the ciphertext $C=\{C_{1}=\left(C_{1,0},C_{1,1},\cdot \;\cdot \;\cdot ,C_{1,n}\right),C_{2},C_{3},C_{4}\}$ is sent from the sender to the cloud server, where $C_{1,i}\in G_{1}$, $C_{2}\in G_{1}$, $C_{3}\in G_{T}$, $C_{4}\in Z_{q}$. Therefore, the communication cost is $\left(n+2\right)\left|G_{1}\right|+\left|G_{T}\right|+\left|Z_{q}\right|=64n+576\;\;\;bytes$. The warrant $w_{ID}=\left\{w_{ID,\;0},w_{ID,1},\cdot \cdot \cdot ,w_{ID,n}\right\}$ is sent from the receiver to the cloud server, where $w_{ID,i}\in G_{1}$. Therefore, the communication cost is $\left(n+1\right)\left|G_{1}\right|=64n+64\;\;\;bytes$. The results of the comparison are listed in Table 4.

From Table 4, we can see that the communication cost of schemes [23,24,25,30,32,33,39] is a fixed value, while that of the proposed scheme and scheme [39] increases linearly with the number of message *n*. From the above analysis, we find that when the message *n* is constant, the public key’s size of the proposed scheme is smaller than those of scheme [39]. As for the size of ciphertext and warrant, the communication cost of the proposed scheme is equal to that of scheme [39]. Thus, the communication cost of the proposed IBE-FET scheme is lower than that of scheme [39].

## 8. Conclusions

In this paper, based on bilinear pairing and secret sharing, we have presented an identity-based encryption with the filtered equality test (IBE-FET) scheme. The security analysis demonstrated that the proposed IBE-FET is OW-ID-CCA secure under the CBDH assumptions in the random oracle model. The performance evaluation and comparison indicate that the proposed IBE-FET achieves greater functionality than most previous schemes and adopts identity-based cryptography which avoids the certificate management issue effectively. In addition, the total computational cost increases linearly with the number of message *n* in all phases. Besides, in terms of communication cost, the proposed scheme is efficient. Therefore, the proposed IBE-FET scheme is more practical.

## Figures and Tables

**Table 1:** Comparison.

Schemes	ET	FET	ID	ROM	Security	Assumption
[23]	✓	✗	✗	✓	OW-CCA	CDH
[24]	✓	✗	✗	✓	OW-CCA,IND-CCA	CDH,DDH
[25]	✓	✗	✗	✓	OW-CCA,IND-CCA	CDH
[30]	✓	✗	✗	✓	OW-CCA,IND-CCA	CONF,CDH
[32]	✓	✗	✓	✓	OW-ID-CCA	CDH
[33]	✓	✗	✓	✓	OW-ID-CCA	CBDH
[39]	✓	✓	✗	✗	IND-CCA	SXDH
The proposed scheme	✓	✓	✓	✓	OW-ID-CCA	CBDH

**Table 2:** Execution time of cryptographic operation.

Cryptographic Operation	Execution Time
Scalar multiplication $T_{E}$	3.7770
Bilinear pairing $T_{P}$	9.0791
Map-to-point hash function $T_{H}$	9.7052

**Table 3:** Computation costs.

Schemes	Encryption	Decryption	Authorization	Equality Test
[23]	$3T_{E}$	$3T_{E}$	⊥	$2T_{P}$
[24]	$4T_{E}$	$2T_{E}$	$3T_{E}$	$4T_{P}$
[25]	$5T_{E}$	$2T_{E}$	⊥	$4T_{P}$
[30]	$6T_{E}$	$5T_{E}$	⊥	$2T_{E}+2T_{P}$
[32]	$6T_{E}+2T_{P}+2T_{H}$	$2T_{E}+2T_{P}+1T_{H}$	$1T_{E}$	$4T_{P}+2T_{H}$
[33]	$2T_{E}+1T_{H}$	$2T_{P}+1T_{H}$	$1T_{H}$	$2T_{E}+4T_{P}+2T_{H}$
[39]	$\left(n+4\right)T_{E}+1T_{H}$	$\left(n+3\right)T_{E}+1T_{P}+1T_{H}$	$\left(n+1\right)T_{E}$	$\left(n+1\right)T_{P}$
The proposed scheme	$\left(n+3\right)T_{E}+2T_{H}+2T_{P}$	$\left(n+1\right)T_{E}+1T_{H}+\left(n+2\right)T_{P}$	$\left(n+1\right)T_{E}$	$\left(n+1\right)T_{P}$

**Table 4:** Communication costs.

Schemes	|PK|	|CT|	|WT|
[23]	$1|G_{1}|=64$ bytes	$3|G_{1}\left|+1\right|Z_{q}|=256$ bytes	⊥
[24]	$2|G_{1}|=128$ bytes	$3|G_{1}\left|+1\right|Z_{q}|=256$ bytes	$3|G_{1}|=192$ bytes
[25]	$2|G_{1}|=128$ bytes	$3|G_{1}\left|+1\right|Z_{q}|=256$ bytes	1$|Z_{q}|=64$ bytes
[30]	$2|G_{1}|=128$ bytes	$5|G_{1}\left|+1\right|Z_{q}|=384$ bytes	⊥
[32]	$2|G_{1}|=128$ bytes	$5|G_{1}\left|+1\right|Z_{q}|=384$ bytes	1$|G_{1}|=64$ bytes
[33]	$2|G_{1}|=128$ bytes	$2|G_{1}\left|+2\right|Z_{q}|=256$ bytes	1$|G_{1}|=64$ bytes
[39]	$\left(n+2\right)|G_{1}\left|+1\right|G_{T}|=64n+512$ bytes	$\left(n+2\right)|G_{1}\left|+1\right|G_{T}|=64n+576$ bytes	$\left(n+1\right)|G_{1}|=64n+64$ bytes
The proposed scheme	$\left(n+2\right)|G_{1}|=64n+128$ bytes	$\left(n+2\right)|G_{1}\left|+1\right|G_{T}\left|+1\right|Z_{q}|=64n+576$ bytes	$\left(n+1\right)|G_{1}|=64n+64$ bytes

## References

[1] Nam T., Pardo T.A. Conceptualizing smart city with dimensions of technology, people, and institutions. Proceedings of the 12th Annual International Digital Government Research Conference on Digital Government Innovation in Challenging Times.

[2] Yu Y., Li Y., Tian J. (2018). Blockchain-based solutions to security and privacy issues in the Internet of Things. IEEE Wirel. Commun..

[3] Su K., Jie L., Hongbo F. Smart city and the applications. Proceedings of the International Conference on Electronics, Communications and Control (ICECC).

[4] Ferraz F.S., Ferraz C.A.G. Smart city security issues: Depicting information security issues in the role of an urban environment. Proceedings of the 7th International Conference on Utility and Cloud Computing (UCC).

[5] Zheng D., Wu A., Zhang Y., Zhao Q. (2018). Efficient and privacy-preserving medical data sharing in Internet of Things with limited computing power. IEEE Access.

[6] Zhang Y., Yang M., Zheng D., Lang P., Wu A., Chen C. (2018). Efficient and secure big data storage system with leakage resilience in cloud computing. Soft Comput..

[7] Catarinucci L., De Donno D., Mainetti L. (2015). An IoT-aware architecture for smart healthcare systems. IEEE Internet Things J..

[8] Demirkan H. (2013). A smart healthcare systems framework. IT Prof..

[9] Acampora G., Cook D.J., Rashidi P. (2013). A survey on ambient intelligence in healthcare. Proc. IEEE.

[10] Zhang Y., Zheng D., Deng R.H. (2018). Security and privacy in smart health: Efficient policy-hiding attribute-based access control. IEEE Internet Things J..

[11] Zhang Y., Lang P., Zheng D., Yang M., Guo R. (2018). A secure and privacy-aware smart health system with secret key leakage resilience. Secur. Commun. Netw..

[12] Zhang Y., Deng R.H., Han G. (2018). Secure smart health with privacy-aware aggregate authentication and access control in Internet of Things. J. Netw. Comput. Appl..

[13] Zhang Y., Zheng D., Guo R., Lan Q. (2018). Fine-grained access control systems suitable for resource-constrained users in cloud computing. Comput. Inf..

[14] Abdalla M., Bellare M., Catalano D. Searchable encryption revisited: Consistency properties, relation to anonymous IBE, and extensions. Proceedings of the Advances in Cryptology-Crypto’05.

[15] Bellare M., Boldyreva A., O’Neill A. Deterministic and efficiently searchable encryption. Proceedings of the Advances in Cryptology-Crypto’07.

[16] Fuhr T., Paillier P. Decryptable searchable encryption. Proceedings of the International Conference on Provable Security.

[17] Boneh D., Di Crescenzo G., Ostrovsky R. Public key encryption with keyword search. Proceedings of the Advances in Cryptology-Crypto’04.

[18] Yau W.C., Heng S.H., Goi B.M. Off-line keyword guessing attacks on recent public key encryption with keyword search schemes. Proceedings of the International Conference on Autonomic and Trusted Computing (ATC).

[19] Ibraimi L., Nikova S., Hartel P. Public-key encryption with delegated search. Proceedings of the International Conference on Applied Cryptography and Network Security (ACNS).

[20] Fang L., Susilo W., Ge C. (2013). Public key encryption with keyword search secure against keyword guessing attacks without random oracle. Inform. Sci..

[21] Baek J., Safavi-Naini R., Susilo W. Public key encryption with keyword search revisited. Proceedings of the International Conference on Computational Science and Its Applications (ICCSA).

[22] Chen R., Mu Y., Yang G. A new general framework for secure public key encryption with keyword search. Proceedings of the Australasian Conference on Information Security and Privacy (ACISP).

[23] Yang G., Tan C.H., Huang Q. Probabilistic public key encryption with equality test. Proceedings of the Cryptographers’ Track at the RSA Conference (CT-RSA).

[24] Tang Q. Towards public key encryption scheme supporting equality test with fine-grained authorization. Proceedings of the Australasian Conference on Information Security and Privacy (ACISP).

[25] Tang Q. (2012). Public key encryption supporting plaintext equality test and user-specified authorization. Secur. Commun. Netw..

[26] Tang Q. (2012). Public key encryption schemes supporting equality test with authorization of different granularity. Int. J. Appl. Cryptogr..

[27] Lu Y., Zhang R., Lin D. Stronger security model for public-key encryption with equality test. Proceedings of the International Conference on Pairing-Based Cryptography.

[28] Ma S., Zhang M., Huang Q. (2014). Public key encryption with delegated equality test in a multi-user setting. Comput. J..

[29] Huang K., Tso R., Chen Y.C. (2015). Pke-aet: Public key encryption with authorized equality test. Comput. J..

[30] Ma S., Huang Q., Zhang M. (2015). Efficient public key encryption with equality test supporting flexible authorization. IEEE Trans. Inf. Forensics Secur..

[31] Lin X.J., Qu H., Zhang X. (2016). Public Key Encryption Supporting Equality Test and Flexible Authorization Without Bilinear Pairings. Cryptology ePrint Archive. http://eprint.iacr.org/2016/277.

[32] Ma S. (2016). Identity-based encryption with outsourced equality test in cloud computing. Inf. Sci..

[33] Wu L., Zhang Y., Choo K.R. (2017). Efficient and secure identity-based encryption scheme with equality test in cloud computing. Future Gener. Comput. Syst..

[34] Zhu H., Wang L., Ahmad H., Niu X. (2017). Key-policy attribute-based encryption with equality test in cloud computing. IEEE Access.

[35] Wang Q., Peng L., Xiong H., Sun J. (2017). Ciphertext-policy attribute-based encryption with delegated equality test in cloud computing. IEEE Access.

[36] Liao Y., Chen H., Li F., Jiang S., Zhou S., Mohammed R. (2018). Insecurity of a key-policy attribute based encryption scheme with equality test. IEEE Access.

[37] Sun J., Bao Y., Nie X., Xiong H. (2018). Attribute-hiding predicate encryption with equality test in cloud computing. IEEE Access.

[38] Huang K., Chen Y.C., Tso R. Semantic secure public key encryption with filtered equality test pke-fet. Proceedings of the 12th International Joint Conference on E-Business and Telecommunications (ICETE).

[39] Huang K., Tso R., Chen Y.C. (2017). Somewhat semantic secure public key encryption with filtered-equality-test in the standard model and its extension to searchable encryption. J. Comput. Syst. Sci..

[40] Boneh D., Franklin M. Identity-based encryption from the Weil pairing. Proceedings of the Advances in Cryptology-Crypto’01.

[41] Amin S.M., Wollenberg B.F. (2005). Toward a smart grid: Power delivery for the 21st century. IEEE Power Energy Mag..

[42] Heydt G.T. (2010). The next generation of power distribution systems. IEEE Trans. Smart Grid..

[43] Alderman J., Farley N., Crampton J. Tree-based cryptographic access control. Proceedings of the 22nd European Symposium on Research in Computer Security (ESORICS).

[44] Alderman J., Crampton J., Farley N. A framework for the cryptographic enforcement of information flow policies. Proceedings of the 22nd ACM on Symposium on Access Control Models and Technologies (SACMAT).

[45] Castiglione A., De Santis A., Masucci B. (2017). Supporting dynamic updates in storage clouds with the Akl-Taylor scheme. Inf. Sci..

[46] Castiglione A., De Santis A., Masucci B. (2016). Key indistinguishability versus strong key indistinguishability for hierarchical key assignment schemes. IEEE Trans. Dependable Secur. Comput..

[47] Yu Y., Ho Au M., Ateniese G., Huang X., Susilo W., Dai Y., Min G. (2017). Identity-based remote data integrity checking with perfect data privacy preserving for cloud storage. IEEE Trans. Inf. Forensics Sec..

[48] Li Y., Yu Y., Susilo W., Min G., Ni J., Choo R. (2019). Fuzzy identity-based data integrity auditing for reliable cloud storage systems. IEEE Trans. Dependable Secur. Comput..

[49] Shamir A. (1979). How to share a secret. Commun. ACM.

[50] Coron J.S. On the exact security of full domain hash. Proceedings of the Advances in Cryptology-Crypto’00.

[51] Ltd S.S. (2019). Multi Precision Integer and Rational Arithmetic Cryptographic Library (MIRACL). http://www.certivox.com/miracl/.

